# The complete chloroplast genome sequence of Chengal (*Neobalanocarpus heimii*, Dipterocarpaceae), a durable tropical hardwood

**DOI:** 10.1080/23802359.2018.1535848

**Published:** 2018-11-21

**Authors:** Shiou Yih Lee, Wei Lun Ng, Muhammad Syahmi Hishamuddin, Rozi Mohamed

**Affiliations:** a Forest Biotech Laboratory, Department of Forest Management, Faculty of Forestry, Universiti Putra Malaysia, Serdang, Selangor, Malaysia;; b China-ASEAN College of Marine Sciences, Xiamen University Malaysia, Sepang, Selangor, Malaysia

**Keywords:** Malay Peninsula, conservation, comparative genomics, phylogenomics, monotypic genus

## Abstract

Known for its durable timber quality, *Neobalanocarpus heimii* (King) Ashton is a highly sought after tree species endemic to the Malay Peninsula. Due to its scarcity and high value, the tree is classified under the IUCN Red List categories of Vulnerable. In this study, we assembled the complete chloroplast (cp) genome of *N. heimii* using data from high-throughput Illumina sequencing. The Chengal cp genome is 151,191 bp in size and includes two inverted repeat regions of 23,721 bp each, which is separated by a large single copy region of 83,801 bp and a small single copy region of 19,948 bp. A total of 130 genes were predicted, including 37 tRNA, 8 rRNA, and 85 protein-coding genes. Phylogenetic analysis placed *N. heimii* within the order Malvales.

The Chengal tree, *Neobalanocarpus heimii* (King) Ashton, is a tropical hardwood found only in the Malay Peninsula. Wood from Chengalis highly durable and is among the strongest in the world (Thomas 1953), making its timber useful and highly valued in heavy construction. It is listed as ‘Vulnerable’ under the IUCN Red List due to decline in its natural habitat and heavy exploitation (Pooma et al. 2017). This monotypic genus is from the huge plant family Dipterocarpaceae. At present, there are uncertainties in the classification of Dipterocarpaceae, compromising the understanding of evolution within the family (Heckenhauer et al. 2017) and hampering conservation efforts of its member species. Currently, there is no complete chloroplast (cp) genome sequence of any Dipterocarpaceae species available in the NCBI GenBank database. We aimed to assemble and characterize *N. heimii*’s cp genome to provide a better understanding on the evolution and genetics of Chengal and other species in the same family.

Total genomic DNA was extracted from fresh leaves collected from a Chengal planted in the arboretum of the Faculty of Forestry, Universiti Putra Malaysia (03°00′09″N, 101°42′19″W). A voucher specimen (FBL05002) is deposited at the Forest Biotechnology Laboratory in the Faculty of Forestry, UPM. A genomic library with an insert size of ∼400 bp was prepared using a TruSeq DNA Sample Prep Kit (Illumina, Beijing) and sequenced on an Illumina HiSeq X Ten platform. Approximately 8 Gb of raw data were generated through pair-end 150 bp sequencing. After removal of adapter sequences, raw reads were fed into the NOVOPlasty (Dierckxsens et al. 2017) for assembly with the *rbc*L gene of *Gossypium hirsutum* (GenBank accession DQ345959) as the seed sequence. The assembled cp genome was annotated using the online annotation tool DOGMA (Wyman et al. 2004) and further corrected manually.

The complete cp genome of *N. heimii* (GenBank accession MH746730) was 151,191 bp in length, consisting of a pair of inverted repeat regions of 23,721 bp each, a large single copy region of 83,801 bp, and a small single copy region of 19,948 bp. A total of 130 genes were annotated, including 37 tRNA, 8 rRNA, and 85 protein-coding genes. The overall GC content of the cp genome was 37.42%.

A phylogenetic analysis was carried out with *N. heimii* and eight other complete cp genomes of species from the order Malvales, comprising of six Malvaceae species (*Bombax ceiba*, *Firmiana major*, *Gossypium arboreum*, *Heritiera angustata*, *Theobroma cacao*, and *Tilia oliveri*) and two Thymelaeaceae species (*Daphne kiusiana* and *Aquilaria yunnanensis*). *Melastoma candidum* from the order Myrtales was included as outgroup. The maximum likelihood tree constructed with RAxML (Stamatakis 2014), implemented in Geneious ver. 10.1 (http://www.geneious.com, Kearse et al. 2012), placed *N. heimii* within the order Malvales (Figure 1).

**Figure 1.**
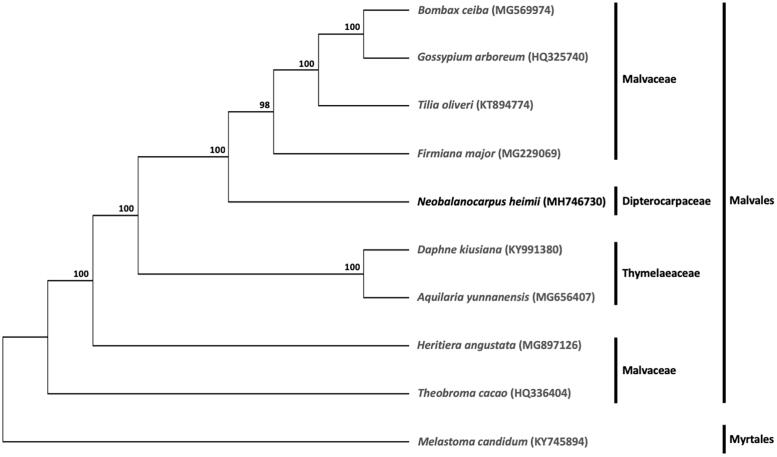
Maximum likelihood tree based on the complete cp genome sequences of nine species from the order Malvales, with *Melastoma candidum* (order Myrtales) as outgroup. Shown next to the nodes are bootstrap support values based on 1000 replicates.

## References

[CIT0001] Dierckxsens N, Mardulyn P, Smits G 2017 NOVOPlasty: de novo assembly of organelle genomes from whole genome data. Nucleic Acids Res. 45:e18. 28204566 10.1093/nar/gkw955 PMC5389512

[CIT0002] Heckenhauer J, Samuel R, Ashton PS, Turner B, Barfuss MHJ, Jang T-S, Temsch EM, Mccann J, Salim KA, Attanayake AMAS, Chase MW 2017 Phylogenetic analyses of plastid DNA suggest a different interpretation of morphological evolution than those used as the basis for previous classifications of Dipterocarpaceae (Malvales). Bot J Linn Soc. 185:1–26.

[CIT0003] Kearse M, Moir R, Wilson A, Stones-Havas S, Cheung M, Sturrock S, Buxton S, Cooper A, Markowitz S, Duran C, et al. 2012 Geneious Basic: an integrated and extendable desktop software platform for the organization and analysis of sequence data. Bioinformatics. 28:1647–1649. 22543367 10.1093/bioinformatics/bts199 PMC3371832

[CIT0004] Pooma R, Barstow M, Newman M 2017 Neobalanocarpus heimii. The IUCN Red List of Threatened Species 2017: e.T32314A2813845. http://oldredlist.iucnredlist.org/details/32314/0

[CIT0005] Stamatakis A 2014 RAxML version 8: a tool for phylogenetic analysis and post-analysis of large phylogenies. Bioinformatics. 30:1312–1313. 24451623 10.1093/bioinformatics/btu033 PMC3998144

[CIT0006] Thomas AR 1953 Malayan timber - Chengal and Balau. Malayan Forester. 16:103–108.

[CIT0007] Wyman SK, Jansen RK, Boore JL 2004 Automatic annotation of organellar genomes with DOGMA. Bioinformatics. 20:3252–3255. 15180927 10.1093/bioinformatics/bth352

